# Nanoparticles from Microalgae and Their Biomedical Applications

**DOI:** 10.3390/md21060352

**Published:** 2023-06-07

**Authors:** Agnieszka Sidorowicz, Giacomo Fais, Mattia Casula, Massimiliano Borselli, Giuseppe Giannaccare, Antonio Mario Locci, Nicola Lai, Roberto Orrù, Giacomo Cao, Alessandro Concas

**Affiliations:** 1 Interdepartmental Centre of Environmental Science and Engineering (CINSA), University of Cagliari, Via San Giorgio 12, 09124 Cagliari, Italy; a.sidorowicz@studenti.unica.it (A.S.); giacomo.fais@unica.it (G.F.); m.casula28@studenti.unica.it (M.C.); antoniom.locci@unica.it (A.M.L.); nicola.lai@unica.it (N.L.); roberto.orru@unica.it (R.O.); giacomo.cao@unica.it (G.C.); 2 Department of Mechanical, Chemical and Materials Engineering, University of Cagliari, Via Marengo 2, 09123 Cagliari, Italy; 3 Department of Ophthalmology, University Magna Grecia of Catanzaro, Viale Europa, 88100 Catanzaro, Italy; mborselli93@gmail.com (M.B.); giuseppe.giannaccare@unicz.it (G.G.)

**Keywords:** microalgae, nanoparticles, synthesis, biomedical, anticancer, antimicrobial

## Abstract

Over the years, microalgae have been a source of useful compounds mainly used as food and dietary supplements. Recently, microalgae have been used as a source of metabolites that can participate in the synthesis of several nanoparticles through inexpensive and environmentally friendly routes alternative to chemical synthesis. Notably, the occurrence of global health threats focused attention on the microalgae application in the medicinal field. In this review, we report the influence of secondary metabolites from marine and freshwater microalgae and cyanobacteria on the synthesis of nanoparticles that were applied as therapeutics. In addition, the use of isolated compounds on the surface of nanoparticles to combat diseases has also been addressed. Although studies have proven the beneficial effect of high-value bioproducts on microalgae and their potential in medicine, there is still room for understanding their exact role in the human body and translating lab-based research into clinical trials.

## 1. Introduction

The increasing human population and life expectancy are causing a change in the leading cause of death, such as heart conditions, cancer, or pulmonary diseases [1]. The shift is pushing the healthcare system to find new solutions to these problems; however, hospitals can be a source of nosocomial infections which are especially dangerous for immunocompromised patients. Another major issue is microbial antibiotic resistance due to antibiotics abuse which can lead to the emergence of life-threatening diseases [2]. 

New promising solutions are introduced by recent developments in nanotechnology which are focused on the manipulation of matter having a characteristic size lower than 100 nm in at least one dimension. The prevailing quantum effect at such a scale can give rise to multiple applications of the products. The prepared materials at the nanoscale might have different features than their bulk equivalents which show the potential for obtaining various properties even within the same element. Several methods are used to synthesize nanoparticles (NPs) such as physical, chemical, or biological routes. Among them, great attention is being paid to biological synthesis due to its low toxicity and biocompatibility, which are crucial for biomedical applications.

One group of organisms used for biological synthesis is microalgae due to their rapid increase in biomass, the independence on arable land, and the abundance of valuable metabolites [3]. Moreover, microalgae can be cultivated also in the wastewater independently from seasonal breaks which is an important economical aspect [4]. The bioactive substances derived from secondary metabolism such as proteins, polysaccharides, lipids, vitamins, and pigments have displayed their great potential for many applications [5] The identified compounds are mainly used for their nutritional value; however, they can participate in the synthesis of various NPs used for biomedical applications. 

Although the use of metabolites in the synthesis of various NPs can result in obtaining highly valuable materials, the mechanism behind it is still unclear [6,7]. In this review, the state of the art related to the participation of microalgal compounds in NPs synthesis is analyzed considering the location of the process (intra- or extracellular). Moreover, recent biomedical applications are taken into account to show the potential of microalgae to be applied in medicine. The recent advancements in the synthesis of NPs using microalgae have been summarized by connecting their synthesis method with resulted performance. Therefore, the work can provide future prospects for the optimization of the synthesis of highly valuable materials using microalgae.

## 2. Biological Synthesis

### 2.1. Microalgal Metabolites

Microalgae are single-celled, photosynthetic organisms found in both marine and freshwater ecosystems. The classification of these organisms is based on the properties such as pigmentation, photosynthetic membrane organization, chemical nature of the photosynthetic storage products, or morphological features [6]. The groups are polyphyletic and highly diverse, with both procaryotic and eucaryotic organisms. The most abundant microalgae are *Cyanophyceae* (blue-green algae), *Bacillariophyceae* (including the diatoms), and *Chlorophyceae* (green algae), with 50,000 estimated existing species, out of which 30,000 species were investigated [6]. Microalgae produce a variety of substances including proteins, carbohydrates, lipids, nucleic acids, vitamins, and minerals [8,9,10]. The cellular content of each group varies depending on the specific strain and their physiological reactions to biotic and/or abiotic factors such as light intensity, photoperiod, temperature, medium composition, and growth phase [11,12]. The typical compounds reported so far participating in the synthesis of nanoparticles are proteins, carbohydrates, and lipids.

**Figure 1:**
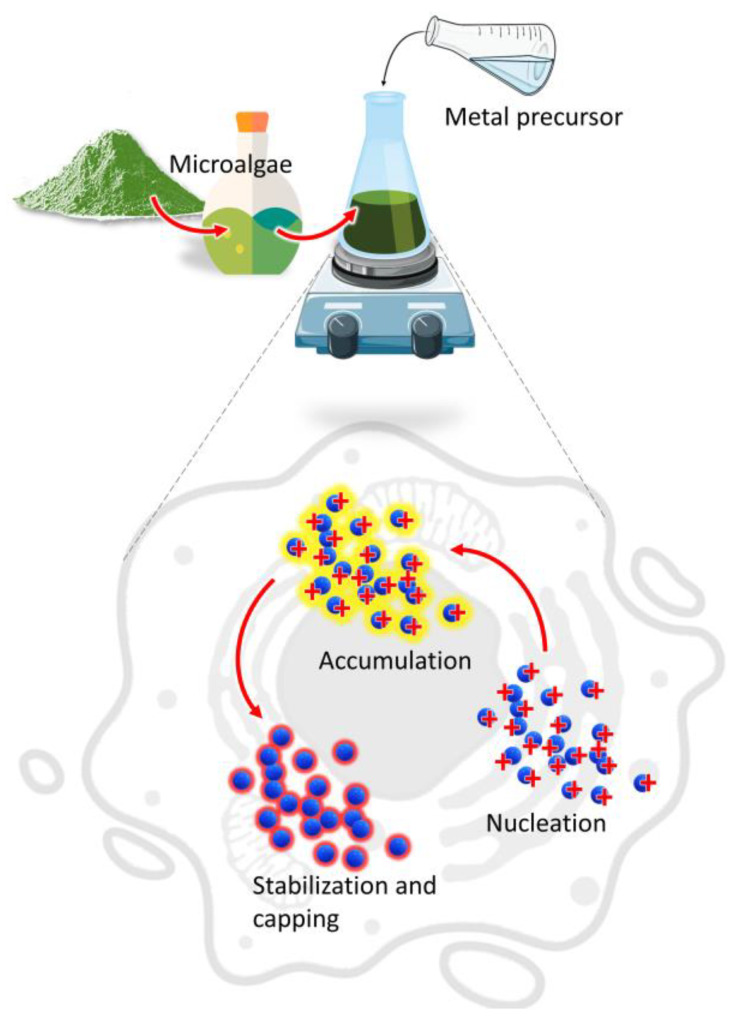
Mechanism of NPs synthesis by microalgae.

The main mechanism involved in the biosynthesis of NPs deals with different metabolites of microalgae that can reduce precursor metal ions into a zerovalent state (Figure 1).

The process involves (i) the activation phase, when the metal ion is reduced, and nucleation occurs, followed by (ii) the growth phase, with an amalgamation of formed unit cells into crystallites which is concluded in (iii) the termination phase, where NPs having different shapes and sizes are thermodynamically stable [13]. Other factors such as temperature, pH, or metal ion concentration could affect the synthesis process; however, the participation of microalgae metabolites is crucial to understand the connection between the synthesis procedure and the properties of the obtained product [14,15]. Moreover, during microalgae cultivation sunlight and carbon dioxide can be acquired from the surroundings while the nutrients could be converted from the wastewater to form biomass which provides the new routes for economic sustainability [16].

#### 2.1.1. Proteins

Proteins are an important component in the structure and metabolism of microalgae. They are an integral part of the cellular membrane and light-harvesting complex as well as they participate as enzymes in numerous catalytic reactions [17,18]. Several species of microalgae are studied due to their high protein content ranging from 42–70% in some cyanobacteria and up to 58% for *Chlorella vulgaris* dry weight [19,20].

The involvement of proteins in the synthesis of nanoparticles is usually investigated using Fourier Transform Infrared Spectroscopy (FTIR) based techniques. The reduction role of the proteins is demonstrated during the oxidation of the −CHO to −COOH group, while NH_2_ groups usually play capping functions through residual amino acids such as cysteine, tyrosine, and tryptophan [21]. In the study by Chokshi et al., the spectra between the prepared extract of *Acutodesmus dimorphus* and prepared Ag NPs were compared, showing the role of amide linkage in the stabilization of Ag NPs by peptides and proteins [22]. The obtained NPs were spherical with 2–20 nm in size. Moreover, the overlapping peaks between the extract and the product suggest their coating properties ensure their stabilization and prevent agglomeration. The surface of NPs might be further modified by sulfonated polysaccharides with proteins that provide a link between nanoparticles and coating molecules [21]. Proteins also possess a strong affinity to bind to metal ions that act as reducing agents [21]. Similar findings have been reported for AgCl NPs from *Chlorella vulgaris* and Ti NPs from marine microalgae *Phaeodactylum tricornutum* [23,24]. Although the exact mechanism of the synthesis is unknown, the statistical experimental design approach has been studied by using response surface methodology for future large-scale production.

#### 2.1.2. Carbohydrates

Similar to proteins, carbohydrates display both structural and metabolic properties. Mono- and oligosaccharides can be found attached to proteins or lipids, forming glycoproteins or glycolipids, while polysaccharides are the major structural component of the cell wall [25]. Moreover, glucose and starch-like energy storage products are obtained during photosynthesis as the primary carbon-containing molecules in microalgae [26]. *Cyanophytes* were reported to accumulate glycogen, while other species form semi-amylopectin [27]. Two glucose polymers, amylopectin, and amylose are starch components of *Chlorophyta*; however, *Rhodophyta* synthesizes a carbohydrate polymer known as floridean starch [10,28]. Diatoms produce chrysolaminarin composed of β(1,3) and β(1,6) linked glucose units which can accumulate around 7% of their total carbon content in the optimal conditions and up to 80% under strong nutrient depletions [29,30].

Carbohydrates are rich in reducing groups, such as hydroxy and carboxy, which can bind and reduce metal atoms, thereby acting as reducing agents. Moreover, due to the supramolecular interactions by inter- and intra-molecular hydrogen bonding, they can stabilize formed nanoparticles and prevent further agglomeration, acting as capping agents [16,31]. The potential of secretory carbohydrates from *C. vulgaris* was tested by the removal of biomass from the culture and used for the synthesis of FeOOH NPs [32]. The synthesis process using carbohydrates was compared with the chemical route with sodium hydroxide acting as a precipitating agent. The carbohydrates were reported to be involved in the nucleation process by chelation of iron ions to prevent monotonic nucleation and limit nuclei size, which is further controlled in the growth phase to inhibit large particle formation. The secretory carbohydrates were described mainly for their reducing properties rather than being capping agents. The obtained NPs were spherical with size range 8–17 nm.

The exopolysaccharides from *Botryococcus braunii* and *Chlorella pyrenoidosa* were also tested for the synthesis of Ag NPs [33]. The polysaccharides performed both reducing and capping functions and were bound to the Ag NPs surface through carboxy and hydroxy groups. A similar size range was reported of 5–15 nm. In a study by Jakhu et al., Au NPs synthesized from *Chlorella* sp. polysaccharides were compared with Au NPs synthesized using citrate to compare their properties [34]. Both products exhibited a controlled size range; however, AuNPs from polysaccharides were stable in the pH range 2–12 while citrate-Au NPs were stable only at basic pH values. The NPs synthesized using polysaccharides were significantly bigger than citrate with sie ranges 30–40 nm and 10–15 nm, respectively. Furthermore, citrate-Au NPs were forming agglomerates in a 30-fold lower concentration of NaCl, further proving the stabilization of the surface of Au NPs by microalgal polysaccharides.

#### 2.1.3. Lipids

Secondary to polysaccharide, lipids function as energy reservoirs as well as structural components of the cell membranes. In microalgae, lipids are mainly composed of (i) neural lipids such as free fatty acids, acylglycerols, and carotenoids, and (ii) polar lipids including phospholipids and galactolipids [35]. The polar lipids fraction can significantly increase during exponential growth; however, during stationary phase when the nutrient availability is limited under stress conditions, they can produce triacylglycerols [36]. The fatty acid content is composed of a mixture of C16 and C18 saturated and unsaturated fatty acids with longer carbon-chains including omega fatty acids. Saturated fats are stored in neutral lipid bodies while unsaturated fats are connected with polar lipids in membranes maintaining membrane fluidity under fluctuating cultivation conditions [37,38]. The overall lipid fraction can represent up to 20–50% of the dry biomass, depending on the microalgal species and cultivation conditions such as nutrient availability, salinity, light intensity, and growth phase [39]. During nutrient depletion, the neutral lipid, and polysaccharide content can increase at the expense of proteins [40]. Lipids receive the greatest attention for extraction followed by the production of biodiesel whereas polyunsaturated fatty acids are used for their nutraceutical value. 

In contrast to water, which is commonly used for synthesis, the involvement of lipids requires the usage of different solvents. Kashyap et al. utilized ethanolic extract to synthesize Ag/AgCl NPs from *Chlorella* sp., *Lyngbya putealis*, *Oocystis* sp., and *Scenedesmus vacuolatus* [41]. During the optimization process, *Oocystis* sp. did not manage to produce NPs while *Chlorella* sp. extract resulted in the synthesis of Ag/AgCl NPs of the smallest size. The study highlighted the role of lipids and proteins along with the hydroxy group stretching movements in the Ag/AgCl NPs formation with the size range of 10–20 nm. In a study by Gusain et al., lipids and carbohydrates were extracted separately from *Acutodesmus obliquus* and used for the synthesis of carbon dots by the microwave thermal method [42]. The products had a size range of 1.2–11 nm. The carbon source did not alter the fluorescence behavior; however, the exact interactions during synthesis were not studied. The optical properties changed with the addition of acetone which demonstrated the potential of using different solvents for the synthesis. The role of lipids is hypothesized mainly as capping agents.

### 2.2. Intracellular Synthesis

Depending on the location where NPs are formed, the corresponding synthesis can be divided into intracellular or extracellular routes. During the former one, live cultures are exposed to the metal precursor, and charged metal ions are transported by negatively charged sites of the cell wall [43]. The trapped ions undergo reduction and form NPs of various sizes and morphologies inside the cell, which require various steps of purification from biomass. 

In a study by Li et al., *Chromochloris zofingiensis* culture was used to prepare Au NPs [44]. The cells after synthesis were characterized showing a peak characteristic for Au NPs in the UV-vis spectrum, which was also confirmed by transmission and scanning electron microscopy findings. The proposed mechanism involves chelation by negatively charged functional groups in the cell wall such as –COOH, –OH, and –OSO_3_H followed by diffusion into the cytosol and reduction by electrons generated from photosynthetic electron transport using enzymes. Other species with different cell wall structures were also investigated for the synthesis mechanism. Instead, *Euglena gracilis* species cell wall possesses a glycoprotein-containing pellicle that allowed metal ions to easily penetrate the cell. On the contrary, the marine microalga *Nitzschia laevis* is composed of a rigid cell wall containing amorphous hydrated porous silica frustule acting as a barrier to reduce ion diffusion into the cells. In addition, Raman spectroscopy was explored as a tool to identify and quantify biomass components in microalgae. 

The effect of Ag/AgCl NPs synthesis on chlorophyll and lipid accumulation was studied on freshwater microalgae *Scenedesmus* sp., showing a decrease of 20–35% after 120 h [45]. However, the cells treated with 0.5 mM AgNO_3_ showed a 75.86% increase in palmitic acid due to the stress induced by Ag/AgCl NPs. Thus, the cells were able to synthesize Ag/AgCl NPs and improve the quality of biodiesel production. Intracellular synthesis was also used to obtain CdSe quantum dots from *C. pyrenoidosa* and *S. obliquus* [46]. First, selenium ions were introduced to the culture to generate selenium precursors within the photosynthetic electron transport system and after 12 h were combined with cadmium ions to form CdSe quantum dots. The algal cells were damaged during the process probably because of precursors reducing enzymatic activity and cell vitality. The intracellular synthesis process requires proper optimization to ensure a high yield while maintaining a low toxicity profile.

### 2.3. Extracellular Synthesis

Extracellular synthesis utilizes either the secreted molecules such as polysaccharides or involves processing of the biomass to produce extract which is utilized for synthesis of NPs [47]. This route is considered more convenient as NPs are easily purified from the solution. In addition, it allows for further modification of metabolites participating in the synthesis by varying adopted solvents, concentration, time, or pH [48,49].

The cell-free filtrate from freshwater microalgae *S. obliquus* culture with different nitrogen sources were used to extracellularly synthesize Ag NPs [50]. The study concentrated on the activity of reductases, nitrogen, and sulfate, on Ag NPs synthesis depending on the composition of the medium. The enzymes are conjugated with electron donors and act as reducing agents. Moreover, the activity influenced not only the yield but also the properties or the obtained Ag NPs especially their size. Consequently, their size inversely correlated antimicrobial activity which demonstrates the importance of metabolites during the synthesis. 

The cell-free *C. vulgaris* culture was investigated for different factors affecting Ag NPs synthesis including time, extract/precursor ratio, temperature, pH, precursor molarity, and incubation conditions [51]. The optimal conditions were maximum incubation time (24 h), silver nitrate/extract ratio (8:2), 37 °C, pH 12, 3 mM silver nitrate, and shaking. The study shows the potential of varying multiple factors during the synthesis of NPs, which might result in products with different properties. In a study by Shalaby et al., algal biomass was processed to obtain an extract which provided metabolites implicated in the synthesis of iron oxide NPs [52]. The synthesis was perfected by varying the ratio of precursor to extract, and the product with the highest absorbance was selected for further application.

## 3. Biomedical Applications of Microalgal NPs

**Figure 2:**
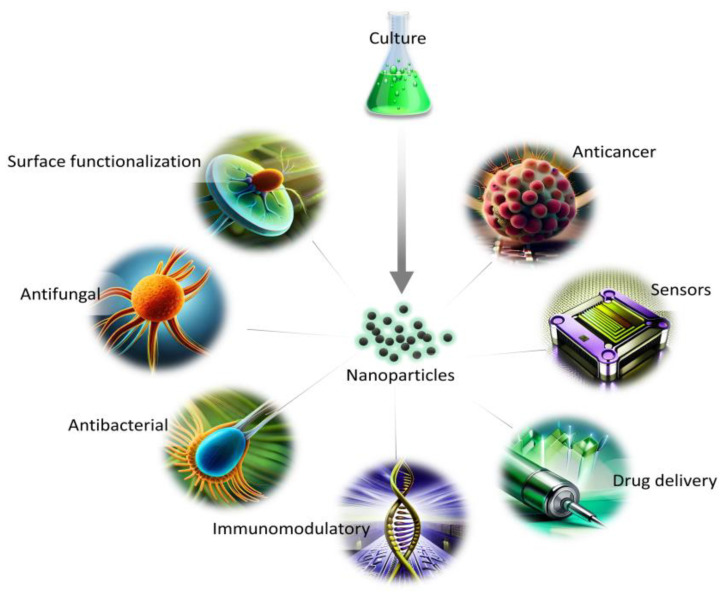
Applications of NPs from microalgae in biomedical fields.

The synthesis route free from toxic waste, as well as economical and environmentally friendly aspects, show the great potential of NPs synthesized from marine and freshwater microalgae for a variety of applications (Figure 2). The presence of naturally occurring biomolecules improves their biocompatibility in comparison with other synthesis routes, and, thus, can be used for biomedical applications. In addition, the growth parameters and metabolites content can be easily altered to obtain a variation in the morphology of NPs for diverse utilization.

The major drawback connected with the synthesis of NPs using a biological approach is their possible wide size distribution or heterogenous morphology. Thus, the obtained product might be difficult to assess in the context of molecular interactions within tissues or organs. The reason behind the differences might be connected with the complexity of molecules participating in the synthesis which reduce the metal ions with varying efficiency. However, the effect can be minimized by utilizing selected classes of secondary metabolites which can also improve the knowledge of their role in the synthesis. Currently, the lack of understanding behind the synthesis mechanism and the long-term effects of NPs are also limiting factors in the biological approach.

### 3.1. Anticancer Activity

**Figure 3:**
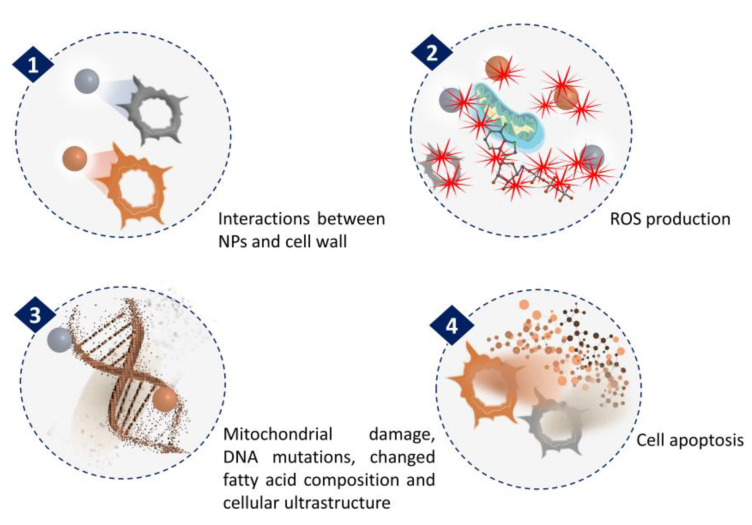
Mechanism of NPs anticancer activity through ROS generation.

The anticancer activity of NPs synthesized from microalgae has been extensively investigated. The general mechanism associated with anticancer activities of NPs is related to ROS generation (Figure 3). In a recent study by Hamida et al., Ag NPs were synthesized using freshwater strain *Coelastrella aeroterrestrica* and their anticancerous activity against four malignant cell lines was compared with chemically synthesized Ag NPs and the anticancer drug 5-fluorouracil [53]. The results showed the highest antiproliferative activity of microalgal Ag NPs against MCF-7, MDA, HCT-116, and HepG2 cell lines with low toxicity against non-cancerous cell lines, compared to the other tested. The activity was attributed to its small size, high stability, less agglomeration, and surface chemistry. However, the mechanistic pathway inside the cancer cell and the pharmacokinetic nature of Ag NPs have yet to be explored.

The influence of various nanocomposites (NCs) synthesized from microalgae on cancer cell lines was also tested. In two separate studies, *S. obliquus* extract was used to conjugate Ag NPs with PtFe_2_O_4_ NPs (PtFe_2_O_4_@Ag) and GaFe_2_O_4_ NPs (GaFe_2_O_4_@Ag) [54,55]. The cytotoxic properties of PtFe_2_O_4_@Ag NCs were related to cross-linking between Pt and DNA to disrupt transcription and replication as well as reactive oxygen species (ROS) generation, lipid peroxidation, and enhanced glutathione (GSH) degradation by Ag NPs. Similarly, Ga in GaFe_2_O_4_@Ag NCs can alter iron metabolism, resulting in the appearance of chromatin fragmentation and apoptotic bodies that induce cell apoptosis. The prepared PtFe_2_O_4_@Ag NCs displayed a more prominent anticancerous activity than the GaFe_2_O_4_@Ag NCs; however, it was lower than cisplatin, a gastric cancer medication used as a control. Furthermore, MgFe_2_O_4_@Ag NCs were synthesized using an extract of *C. vulgaris* showing anticancer activity through a similar apoptotic pathway [56]. The role of MgO in the NCs was attributed to the enhancement of the magnetic properties and disruption of the cell membrane. Further studies are needed to enhance NCs activity and describe their effects in various cancerous and non-cancerous cell lines. Other reported NPs synthesized using microalgae tested for anticancer activity are presented in Table 1.

Microalgae can serve as a source of valuable compounds that not only take part in the synthesis of anticancer drugs but also exhibit anticancer properties themselves. However, their proper action requires maintaining their structure which could be damaged due to chemical or physical factors. In a study by İnan et al., the marine microalga *C. variabilis* and *C. pyrenoidosa* oil extracts were encapsulated into NPs using an electrospraying technique [57]. Encapsulated oil extracts showed higher biocompatibility, while only *C. variabilis* oil extract showed improved anticancer properties compared to the non-encapsulated form. The activity changed in a dose-dependent manner with observed changes in the morphology of the cells. The research on optimizing encapsulation techniques could lead to the development of novel anticancer drugs of microalgal origin.

**Table 1:** Anticancer activity of NPs synthesized from microalgae.

Types of NPs	Microalgae Species Used	General Environment	Size and Morphology of NPs	Tested Cancerous Cell Lines	Ref.
Silver NPs (Ag NPs)	*Arthrospira platensis*	marine, freshwater	2.23–14.68 nm, spherical	A549, HCT, Hep2 and WISH	[58]
Copper NPs (CuO NPs)	*Arthrospira platensis*	marine, freshwater	3.75–12.4 nm, spherical	A549, HCT, Hep2 and WISH	[58]
Silver NPs (Ag NPs)	*Arthrospira platensis*	marine, freshwater	30 nm, spherical	A-549, MCF-7	[59]
Au/cellulose nanocomposite	*Chlorella vulgaris*	freshwater	113–203 nm, spherical	A-549	[60]
Gold NPs (Au NPs)	*Dunaliella salina*	marine	22.4 nm, spherical	MCF-7	[61]
Carbon quantum dots	*Pectinodesmu* sp.	freshwater	67 nm, spherical	HCC 1954, HCT 116	[62]
Silver NPs (Ag NPs)	*Trichodesmium erythraeum*	marine	26.5 nm, cubical	MCF-7, He La	[63]
Silver NPs (Ag_2_O/|AgO NPs)	*Oscillatoria* sp.	Freshwater	4.42–48.97 nm, quasi-spherical	CaCo-2, HeLa	[64]

### 3.2. Biomedical Sensor

Algal-synthesized NPs have shown the potential to be utilized for the detection of dopamine, which plays an essential role in renal, central nervous, cardiovascular, and hormonal regulation. Moreover, it acts as a neurotransmitter in reward and movement regulation in the brain and its abnormal concentrations can cause neurological disorders. The study by Huang et al. utilized ethanolic extract of the marine microalga *Spirulina* to synthesize Au NPs with 11–14 nm in size for dopamine detection [65]. The proteins and polysaccharides from the extract participated in the synthesis as reducing and capping agents with an abundance of –OH and –COOH groups on the Au NPs surface. The interactions between functional groups of dopamine cause changes in Au NPs’ surface allowing dopamine to be adsorbed, thus forming additional hydrogen, ester, and amide bonds with other neighboring Au NPs. In that way, dopamine can act as a linkage between Au NPs with a new enhanced plasmon resonance absorption peak changing the color of the solution from wine red to blue-black. The set-up was tested to evaluate selectivity and anti-interference showing specificity in the presence of various components such as amino acids, salts, and glucose. However, when the Ca^2+^ content was above 200 μM, it could form complexes with the oxygen of the –COOH group causing Au NPs aggregation, which can be thus prevented by keeping Ca^2+^ concentration at lower levels. When tested in human urine, the method was proven to be accurate and precise with a simple detection protocol. 

Another compound that could be detected by NPs synthesized by microalgae is atropine, a tropane alkaloid used to treat low heart rate (bradycardia), an overdose of cholinergic drugs, and cholinergic poisoning, as well as to help reduce saliva, mucus, or other secretions during surgery [66]. The marine microalga *S. platensis* biomass was used in a process to obtain Ag NPs on their surface as a coating material with an average size of 59 nm. The product was placed on the electrode with and without a binder to test its properties. The results revealed high electrocatalytic activity in atropine determination with response fluctuation by a change in pH value. The selectivity, stability, and reproducibility test proved the potential of the material as a stable sensor. In addition, the performance was confirmed by using atropine sulfate ampoule and water as real samples further elaborating the high accuracy and recovery rate of the prepared electrode.

In addition to neurotransmitters or pharmaceuticals, the glucose level can also be monitored by NPs from microalgae [67]. The biomass of *C. vulgaris* was processed using the hydrothermal method, acid hydrolysis assisted by ultrasonics, and was followed by the hydrothermal method to obtain carbon dots. After each hydrothermal treatment, the carbon dots were collected to compare their synthesis method with properties. Acidic hydrolysis was considered crucial to degrade starch and cellulose to reduce sugars to increase yield and prevent interactions between Fe^2+^ and residual –NH groups on the surface. In addition, low pH helped to prevent quenching during the fluorescence response of carbon dots. The sensing mechanism was tested for Fe^3+^ and H_2_O_2_ and then applied to determine the glucose level in blood samples based on the reaction of the glucose oxidase enzyme and the quenching of fluorescence under optimized conditions. The results revealed high sensitivity and selectivity of the sensor obtained with potential use in diagnostics.

### 3.3. Drug Delivery

Targeted drug delivery is a strategy to selectively administer pharmaceuticals into specific areas to maximize its efficacy while avoiding side effects. In a study by Wang et al., microrobots were prepared for drug loading, targeted delivery, and chemo-photothermal therapy [68]. First, *S. platensis* cells were used as a template for core–shell-structured Pd@Au NPs synthesis by electroless deposition to act as photothermal conversion agents to allow laser-triggered degradation to release the drug. Additionally, Fe_3_O_4_ NPs were deposited on the surface via a sol–gel process for the magnetic actuation of the material. The anticancer drug doxorubicin was loaded on the prepared structure to allow their chemotherapeutic effect. The obtained microrobots exhibited significant propulsion under a magnetic field and can be structurally disassembled into individual components under laser irradiation to be used for pH- and laser-triggered drug release. Moreover, Au and Fe_3_O_4_ NPs can be utilized as CT and MR imaging contrast agents, demonstrating the real-time monitoring of the treatment. Considering their small size, the authors suggest oral administration for gastrointestinal cancer therapy.

Doxorubicin-loaded microrobots were also synthesized using the marine microalga *Thalassiosira weissflogii* as a template to substitute mesoporous silica NPs [69]. The magnetic Fe_3_O_4_ NPs were adhered to the template surface for the magnetic actuation properties. Using an external magnetic field, the movement of microrobots can be controlled in trajectories by changing the frequency, allowing microrobots to move through channels of various diameters. The clustering behavior enables the microrobots to carry high-load to the target position with subsequent release in a pH-sensitive manner. The microrobots were tested on MCF-7 human breast cancer cells demonstrating their efficiency. Further studies in vivo are required to test their application. 

In addition, the marine microalga *T. weissflogii* was utilized in a separate study as a template for the delivery of curcumin-loaded drugs for anticancer and antibacterial properties [70]. The nanoporous architecture of *T. weissflogii* frustules provided a cage-like structure for curcumin adsorption which was stabilized by interactions of functional groups. The potential of *T. weissflogii* to be used for drug delivery systems shows its future application for the treatment of a wide range of diseases.

Microalgae can act not only as a template for drug delivery but can also be a source of valuable molecules that display therapeutic activities. Freshwater microalgae *Botryococcus braunii* and *Microcystis aeruginosa* oil, rich in polyunsaturated fatty acids, were loaded into NP electrosprayed with alginate/polyvinylidene (PVA) and tested for antibacterial activity with stabilized release [71]. The encapsulation protected bioactive and antioxidant properties of the oil which shows the potential for their storage and use for commercial application.

### 3.4. Immunomodulatory Action

The study by Chandrarathna et al. investigated the effect of pectin, a polysaccharide isolated from the marine strain *Spirulina maxima* [72]. The nano form of the compound was obtained through sonication to avoid possible modifications from the addition of chemicals. The material was tested on the mice model in comparison with non-sonicated pectin, with an average particle size of 64.11 nm and 152.90 nm for sonicated and original pectin, respectively. The results revealed increased weight gain in the nano-pectin-treated mice due to the improved digestibility and availability of nutrients with the small particle size. In addition, small particle size provided a higher surface area for microbial growth in the intestines than the original longer pectin molecules. The mice treated with nano-pectin displayed an increased density of goblet cells in the gut barrier which blocks the access of pathogenic microbes to the gut epithelium as well as showed higher expression of intestinal alkaline phosphatases, which provide an anti-inflammatory effect. It was assumed that in order to better understand the interactions between pectin and gut microbiome long-term studies are required.

The immunomodulatory effect of nano-pectin from *S. maxima* was also tested in zebrafish both in vitro and in vivo [73]. The low toxicity of nano-pectin was hypothesized to be due to the morphological properties as well as the surface functionalization. In addition, the transcriptional analysis of immune-related genes was performed to further describe the immunomodulatory action. The results showed increased expression of cytokines and antioxidants that participate in the innate immune response. Altogether, nano-pectin was crucial for stress tolerance and anti-inflammatory actions on the molecular level for disease resistance. Although the wound healing activity was not remarkable, nano-pectin was hypothesized to engage the immune system during the process. The studies on mice and zebrafish show the potential of nano-pectin to regulate the immune system response in different organisms; however, further investigations are required to understand the underlying mechanisms behind those actions.

Polysaccharides from a different marine species, *Arthrospira fusiformis*, were also tested for the synthesis of Ag NPs and their immunomodulatory properties [74]. The prepared material was tested in vitro on *Pseudomonas aeruginosa* as well as in vivo on *P. aeruginosa*-infected rat models. The Ag NPs were on average around 10 nm in size with homogenous distribution. The findings showed a strong antibacterial effect due to the breakage of the outer membrane of *P. aeruginosa*, affecting cell permeability with the disruptions created called “pits” that lead to cell lysis, as investigated by in vitro studies. The algal coating was suggested to interact with human serum protein by slightly reducing its concentration resulting in increased cellular uptake and intracellularly killing of bacteria. Moreover, Ag NPs used as wound dressing of P. aeruginosa-infected areas enhanced the wound healing by cytokine modulation and reducing the inflammation. The proposed mechanism involves the liberation of Ag^+^ from Ag NPs and their sequestration by H_2_S-synthesizing enzymes in the macrophage resulting in the formation of Ag_2_S and lowering the inflammation. After seven days of treatment, the tissue structure in rats was restored, which shows the potential of Ag NPs from algae for future treatments which should be assessed during clinical trials.

### 3.5. Antibacterial Activity

Antibiotic resistance can be counted among the worst threats to human health worldwide. Overuse of antibiotics leads to the emergence of multidrug bacterial strains, which are difficult to target using currently available medicaments. Therefore, as an alternative, the use of NPs has been proposed as novel antibacterial agents with superior bactericidal activity. The NPs obtained can alter the cell wall and membrane, penetrate the cytosol, and generate reactive oxygen species (ROS), leading to further damage to enzymes, lipids, and DNA [75]. The biomolecules on the NP’s surface can enhance the antibacterial activity, while their exact role is not well understood. The activity targets both Gram-positive and Gram-negative bacteria including multidrug-susceptible as well as multidrug-resistant strains [75]. However, the antibacterial activity can vary between different tested bacterial strains of the same species probably due to the horizontal gene transfer [76]. As a result, bacteria may acquire genome islands encoding enzymes responsible for antimicrobial resistance to NPs. The role of NPs synthesis and morphology on their activity is presented in Table 2. The antibacterial activity was mainly tested using Ag NPs, however, other NPs such as Au NPs or ZnO NPs are also studied for their properties. Moreover, different microalgal species used for the synthesis result in various morphologies of the prepared NPs which further signifies the potential of microalgae to obtain antibacterial agents. 

### 3.6. Antifungal Activity

Emerging resistance to antifungal drugs with their limited availability becomes a growing public health concern, leading to an increase in fungal infections. Moreover, fungal infections can rapidly spread in hospitals, especially between immunocompromised patients [90]. Therefore, increasing attention is being paid to satisfy the need to develop new and effective antifungal agents. So far, NPs have demonstrated excellent fungicidal activity against several fungal species, as shown in Table 3. The antifungal activity was studied for a variety of NPs, including Au NPs, Fe_3_O_4_ NPs, Ag NPs, or Co(OH)_2_ nanoflakes with various size ranges and morphologies. In addition, microalgae can contain molecules with fungicidal activity and if they also participate in the synthesis as reducing or capping agents, it would offer synergistic antifungal action [91].

### 3.7. Functionalization to Reduce Toxicity

Microalgae are also a source of valuable compounds which can be further stabilized on the NPs’ surface. The study by Torrez-Diaz et al. proposed a method to stabilize *Chlorella* freshwater microalgae peptide (VECYGPNRPQF) on the Au NPs surface [96]. The NPs of Au were obtained through the chemical method of citrate reduction, and then peptide solution was added to the flask. The average product size was 15 nm in diameter and spherical in size with high stability after removing stabilizing agents and centrifuge runs. Peptide-functionalized Au NPs showed almost three times higher antioxidant activity than non-functionalized Au NPs, as well as decreased marine ecosystem toxicity, which could be linked with the increased stability of functionalized Au NPs. The effect of the peptide on the marine organisms was hypothesized as either adaptation or usage as a nutrient in stressful environments.

In a study by Rudi et al. Ag NPs from the commercial source were functionalized by adding to the culture of marine microalgae *S. platensis* and extraction from the biomass [97]. The product was spherical with 8–20 nm in size and administered to rats in comparison to PEG-Ag NPs. A greater concentration of silver was observed in the liver; however, the presence of silver in the brain tissues confirms the ability of Ag NPs to penetrate the blood–brain barrier. The *Spirulina*-functionalized Ag NPs were excreted from all organs except the brain while non-modified PEG-Ag NPs were also present in the liver. However, in a study by El-Deeb et al., upon treatment, the level of liver enzymes ALT and AST increased, while the concentration of urea and albumin remained normal, suggesting the interactions between Ag NPs mainly in the liver. The complexity of interactions between NPs and various organs should be assessed in a long-term study to understand the underlying molecular mechanisms.

The Se NPs synthesized using different amounts of marine microalgae *S. platensis* polysaccharide extract were tested to assess their cytotoxicity against several cancer cell lines [98]. Increased concentration of polysaccharides decreased sizes of the prepared Se NPs to 20–50 nm with more homogeneous size distribution than without added polysaccharides. Moreover, Se NPs prepared from *S. platensis* polysaccharides were stable for at least three months with an almost nine-fold increase in uptake by the cells. Subsequently, enhanced uptake improved the anti-cancer activity through apoptosis induction. In addition, the material showed selectivity between cancer and normal cell lines also displaying the potential for cancer chemoprevention. 

In a separate study, Se NPs were functionalized with different amounts of phycocyanin, pigment purified from marine microalgae *Spirulina* sp., against insulinoma cells [99]. The phycocyanin showed a similar tendency for the size and size distribution of Se NPs as that of polysaccharides; however, increasing the phycocyanin content increased the shell diameter surrounding Se NPs. Thus, the phycocyanin dosage was optimized, as smaller NPs are up-taken easily due to their larger surface area. The functionalized Se NPs showed protective action against intracellular ROS overproduction, mitochondria fragmentation, and activation of enzymes leading to cell apoptosis, induced by palmitic acid. The cytoprotective activity of functionalized Se NPs show their potential against diseases related to pancreatic islet damage.

## 4. Conclusions and Future Prospectives

Undoubtedly, microalgae serve as excellent candidates for the synthesis of a variety of NPs due to their rich content of secondary metabolites acting as capping and reducing agents. However, the process can suffer from several limitations such as low yield, the need for optimization of conditions, or the amount of time to complete the synthesis. The exact mechanism involving the action of metabolites is needed to describe NPs production. The wide range of NPs synthesized from microalgae applied in the biomedical sector shows the potential of the metabolites to influence the physicochemical properties of NPs. Further research is required to address the issues of kinetics, cell viability, and yield with their effect on the properties of NPs synthesized by conventional methods and by using microalgae.

As far as the future implications are concerned, it can be assumed that the alteration of synthesis conditions might also lead to extending the knowledge of the role of metabolites on the obtained NPs. Various species of microalgae and precursors can be tested for their application in the biomedical field. Moreover, diverse techniques applied in the extract preparation, targeting certain classes of metabolites might explain their role in the synthesis. Therefore, future studies on the synthesis of NPs using microalgae can lead not only to the optimization of the process but also to the conceptual understanding of the connection between synthesis, properties, and activity of NPs.

## Figures and Tables

**Table 2:** Antibacterial activity of NPs synthesized from microalgae.

Types of NPs	Microalgae Species Used	General Environment	Size and Morphology of NPs	Bacteria Species Tested	Ref.
Gold NPs (AuNPs)	*Arthrospira platensis*	marine, freshwater	5 nm, spherical	*S. aureus*, *B. subtilis*	[77]
Gold NPs (AuNPs)	*Neodesmus pupukensis (MG257914)*	freshwater	5–34 nm, circular	*Pseudomonas* sp., *Serratia marcescens*	[78]
Silver NPs (AgNPs)	*Chlorococcum humicola (IMMTCC-17)*	freshwater	2–16 nm, spherical	*E. coli (ATCC-1105)*	[79]
Silver NPs (AgNPs)	*Scenedesmus* sp. *(IMMTCC-25)*	marine, freshwater	5–10 nm, spherical	*S. cutans*, *E. coli*	[80]
Silver NPs (AgNPs)	*Chlorella vulgaris* sp.	freshwater, terrestrial	7 nm, spherical	*S. aureus*, *E. coli*	[81]
Silver NPs (AgNPs)	*Chroococcus minutus*	freshwater	crystalline	*E. coli*, *S. aureus*, *P. aeruginosa*,	[82]
Silver NPs (AgNPs)	*Oscillatoria limnetica*	freshwater	3.30–17.97 nm, spherical/anisotropic	*E. coli*, *B. cereus*	[83]
Silver NPs (AgNPs)	*Oscillatoria princeps*	marine, brackish, freshwater,	3.30–17.97 nm, spherical	*S. aureus*, *S. pyogenes*, *E. coli*,	[84]
Silver NPs (AgNPs)	*Anabaena* sp. *66-2*, *Cylindrospermopsis* sp. *USC-CRB3*, *Synechocystis* sp. *48-3*, *B. braunii*,	marine, brackish, freshwater	13–25 nm, spherical/elongated	*B. megaterium*, *E. coli*, *B. subtilis*, *M. luteus*, *P. aeruginosa*, *S. aureus*	[85]
Silver NPs (AgNPs)	*Chlorella pyrenoidosa NCIM 2738*	freshwater	8 nm, irregular	*K. pneumoniae*, *A. hydrophila*, *Acenetobacter* sp., *S. aureus*	[86]
Silver NPs (AgNPs)	*Chlorella vulgaris* sp. *(C. vulgaris)*	freshwater	1.6–34.4 nm, spherical	*Staphilococcus Aureus*, *Klebsiella Pneumonia*	[23]
Silver NPs (AgNPs)	*Neodesmus pupukensis (MG257914)*	freshwater	52–179 nm, spherical	*Pseudomonas aeruginosa*, *E. coli*, *K. Pneumoniae*, *S. marcescens*	[78]
Silver NPs (AgNPs)	*Spirogyra varians*	freshwater	17.6 nm, spherical	*B. cereus*, *P. aeruginosa and Klebsiella*, *S. aureus*, *L. monocytogenes*, *E. coli*	[87]
Silver NPs (AgNPs)	*Coelastrella aeroterrestrica*	freshwater	14.5 nm, hexagonal	*Staphylococcus aureus*, *Streptococcus pyogenes*, *Bacillus subtilis*, *Escherichia coli*, *Pseudomonas aeruginosa*	[53]
Silver NPs (AgNPs)	*Limnothrix* sp. *37-2-1*	freshwater	31.86 nm, elongated	*B. megaterium*, *E. coli*, *B. subtilis*, *M. luteus*, *P. aeruginosa*, *S. aureus*	[85]
Silver NPs (AgNPs)	*Anabaena* sp. 66-2	brackish	24.13 nm, irregular	*B. megaterium*, *E. coli*, *B. subtilis*, *M. luteus*, *P. aeruginosa*, *S. aureus*	[85]
Silver NPs (AgNPs)	*Synechocystis* sp. 48-3	marine, brackish	14.64 nm, irregular	*B. megaterium*, *E. coli*, *B. subtilis*, *M. luteus*, *P. aeruginosa*, *S. aureus*	[85]
Silver NPs (AgNPs)	*Botryococcus braunii*	freshwater	15.67 nm, spherical	*B. megaterium*, *E. coli*, *B. subtilis*, *M. luteus*, *P. aeruginosa*, *S. aureus*	[85]
Silver NPs (AgNPs)	*Coelastrum* sp. 143-1	freshwater	19.28 nm, spherical	*B. megaterium*, *E. coli*, *B. subtilis*, *M. luteus*, *P. aeruginosa*, *S. aureus*	[85]
Silver NPs (AgNPs)	*Limnothrix* sp. 37-2-1	freshwater	25.65 nm, spherical and elongated	*B. megaterium*, *E. coli*, *B. subtilis*, *M. luteus*, *P. aeruginosa*, *S. aureus*	[85]
Silver NPs (AgNPs)	*Arthrospira platensis*	marine, freshwater	13.85 nm, spherical	*B. megaterium*, *E. coli*, *B. subtilis*, *M. luteus*, *P. aeruginosa*, *S. aureus*	[85]
Zinc oxide NPs (ZnO)	*Chlorella vulgaris* sp. *(C. vulgaris)*	freshwater	150 nm crystalline structure/21 nm rod-like appearance	*Staphylococcus aureus*, *Enterococcus faecalis*, *Escherichia coli*, *Pseudomonas aeruginosa*	[88]
Zinc oxide NPs (ZnO)	*Arthrospira platensis*	marine, freshwater	30.0–55.0 nm, spherical	*Bacillus subtilis*, *Staphylococcus aureus*, *Pseudomonas aeruginosa*, *Escherichia coli*	[89]

**Table 3:** Antifungal activity of NPs synthesized from microalgae.

Types of NPs	Microalgae Species Used	General Environment	Size and Morphology of NPs	Species of Fungi Tested	Ref.
Cobalt hydroxide NPs (Co(OH)_2_ NMs)	*Arthrospira platensis*	marine, freshwater	3.52 nm, nanoflake	*C. albicans*, *C. glabrata*, *C. krusei.*	[92]
Cobalt oxide NPs (Co_3_O_4_ NMs)	*Arthrospira platensis*	marine, freshwater	13.28 nm, nanoflake	*C. albicans*, *C. glabrata*, *C. krusei.*	[92]
Gold NPs (AuNPs)	*Neodesmus pupukensis (MG257914)*	freshwater	5–34 nm, circular shape	*A. niger*, *A. fumigatus*, *A. flavus*, *F. solani C. albicans*	[78]
Gold NPs (AuNPs)	*Chlorella sorokiniana*	freshwater	20–40 nm, spherical	*C. tropicalis*, *C. glabrata*, and *C. albicans*	[93]
Gold NPs (AuNPs)	*Chlorella Vulgaris*	freshwater	2–10 nm, spherical	*C. albicans*	[94]
Iron oxide NPs (Fe_3_O_4_ NPs)	*Chlorella K01*	freshwater	50–100 nm, spherical	*Fusarium oxysporum*, *Fusarium tricinctum*, *Fusarium maniliforme*, *Rhizoctonia solani*, *and Phythium* sp.	[95]
Silver NPs (AgNPs)	*Arthrospira platensis*	marine, freshwater	9.72 nm (before calcination)/26.01 nm (after calcination), oval-shaped	*C. albicans*, *C. glabrata*, *C. krusei.*	[92]
Titanium dioxide NPs (TIO_2_ NPs)	*Arthrospira platensis*	marine, freshwater	4.81 nm (before calcination)/4.62 nm (after calcination), spherical-shaped	*C. albicans*, *C. glabrata*, *C. krusei.*	[92]
Zinc oxide NPs (ZnO)	*Arthrospira platensis*	marine, freshwater	≈30.0 to 55.0 nm, spherical	*C. albicans*	[89]

## Data Availability

Not applicable.

## References

[1] Ritchie H., Roser M. Causes of Death, Our World in Data. https://ourworldindata.org/causes-of-death.

[2] Inda-Díaz J.S., Lund D., Parras-Moltó M., Johnning A., Bengtsson-Palme J., Kristiansson E. (2023). Latent Antibiotic Resistance Genes Are Abundant, Diverse, and Mobile in Human, Animal, and Environmental Microbiomes. Microbiome.

[3] Santiago-Díaz P., Rico M., Rivero A., Santana-Casiano M. (2022). Bioactive Metabolites of Microalgae from Canary Islands for Functional Food and Feed Uses. Chem. Biodivers..

[4] Ray A., Nayak M., Ghosh A. (2022). A Review on Co-Culturing of Microalgae: A Greener Strategy towards Sustainable Biofuels Production. Sci. Total Environ..

[5] Ibrahim T.N.B.T., Feisal N.A.S., Kamaludin N.H., Cheah W.Y., How V., Bhatnagar A., Ma Z., Show P.L. (2023). Biological Active Metabolites from Microalgae for Healthcare and Pharmaceutical Industries: A Comprehensive Review. Bioresour. Technol..

[6] Cai Y., Lim H.R., Khoo K.S., Ng H.-S., Cai Y., Wang J., Tak-Yee Chan A., Show P.L. (2021). An Integration Study of Microalgae Bioactive Retention: From Microalgae Biomass to Microalgae Bioactives Nanoparticle. Food Chem. Toxicol..

[7] Maqbool Q., Yigit N., Stöger-Pollach M., Ruello M.L., Tittarelli F., Rupprechter G. (2023). Operando Monitoring of a Room Temperature Nanocomposite Methanol Sensor. Catal. Sci. Technol..

[8] Soru S., Malavasi V., Caboni P., Concas A., Cao G. (2019). Behavior of the Extremophile Green Alga Coccomyxa Melkonianii SCCA 048 in Terms of Lipids Production and Morphology at Different PH Values. Extremophiles.

[9] Soru S., Malavasi V., Concas A., Caboni P., Cao G. (2019). A Novel Investigation of the Growth and Lipid Production of the Extremophile Microalga Coccomyxa Melkonianii SCCA 048 under the Effect of Different Cultivation Conditions: Experiments and Modeling. Chem. Eng. J..

[10] Tsvetanova F., Yankov D. (2022). Bioactive Compounds from Red Microalgae with Therapeutic and Nutritional Value. Microorganisms.

[11] Gao P., Guo L., Gao M., Zhao Y., Jin C., She Z. (2022). Regulation of Carbon Source Metabolism in Mixotrophic Microalgae Cultivation in Response to Light Intensity Variation. J. Environ. Manag..

[12] Udayan A., Pandey A.K., Sirohi R., Sreekumar N., Sang B.I., Sim S.J., Kim S.H., Pandey A. (2022). Production of Microalgae with High Lipid Content and Their Potential as Sources of Nutraceuticals. Phytochem. Rev..

[13] Khan F., Shahid A., Zhu H., Wang N., Javed M.R., Ahmad N., Xu J., Alam M.A., Mehmood M.A. (2022). Prospects of Algae-Based Green Synthesis of Nanoparticles for Environmental Applications. Chemosphere.

[14] Dinc S.K., Vural O.A., Kayhan F.E., San Keskin N.O. (2022). Facile Biogenic Selenium Nanoparticle Synthesis, Characterization and Effects on Oxidative Stress Generated by UV in Microalgae. Particuology.

[15] Aratboni H.A., Rafiei N., Allaf M.M., Abedini S., Rasheed R.N., Seif A., Barati B., Wang S., Morones-Ramírez J.R. (2023). Nanotechnology: An Outstanding Tool for Increasing and Better Exploitation of Microalgae Valuable Compounds. Algal Res..

[16] Chan S.S., Low S.S., Chew K.W., Ling T.C., Rinklebe J., Juan J.C., Ng E.P., Show P.L. (2022). Prospects and Environmental Sustainability of Phyconanotechnology: A Review on Algae-Mediated Metal Nanoparticles Synthesis and Mechanism. Environ. Res..

[17] Zittelli G.C., Lauceri R., Faraloni C., Margarita A., Benavides S., Torzillo G. (2023). Valuable Pigments from Microalgae: Phycobiliproteins, Primary Carotenoids, and Fucoxanthin. Photochem. Photobiol. Sci..

[18] Kumar R., Hegde A.S., Sharma K., Parmar P., Srivatsan V. (2022). Microalgae as a Sustainable Source of Edible Proteins and Bioactive Peptides—Current Trends and Future Prospects. Food Res. Int..

[19] Yucetepe A. (2022). A Combination of Osmotic Shock and Ultrasound Pre-Treatments and the Use of Enzyme for Extraction of Proteins from *Chlorella vulgaris* Microalgae: Optimization of Extraction Conditions by RSM. J. Food Meas. Charact..

[20] García-Gómez C., Márquez-Reyes J.M., Vidales-Contreras J.A., Nápoles-Armenta J., Luna-Maldonado A.I. (2022). The Use of Microalgae and Cyanobacteria for Wastewater Treatment and the Sustainable Production of Biomass. Omics for Environmental Engineering and Microbiology System.

[21] Shankar P.D., Shobana S., Karuppusamy I., Pugazhendhi A., Ramkumar V.S., Arvindnarayan S., Kumar G. (2016). A Review on the Biosynthesis of Metallic Nanoparticles (Gold and Silver) Using Bio-Components of Microalgae: Formation Mechanism and Applications. Enzyme Microb. Technol..

[22] Chokshi K., Pancha I., Ghosh T., Paliwal C., Maurya R., Ghosh A., Mishra S. (2016). Green Synthesis, Characterization and Antioxidant Potential of Silver Nanoparticles Biosynthesized from de-Oiled Biomass of Thermotolerant Oleaginous Microalgae Acutodesmus Dimorphus. RSC Adv..

[23] da Silva Ferreira V., ConzFerreira M.E., Lima L.M.T.R., Frasés S., de Souza W., Sant’Anna C. (2017). Green Production of Microalgae-Based Silver Chloride Nanoparticles with Antimicrobial Activity against Pathogenic Bacteria. Enzyme Microb. Technol..

[24] Caliskan G., Mutaf T., Agba H.C., Elibol M. (2022). Green Synthesis and Characterization of Titanium Nanoparticles Using Microalga, *Phaeodactylum tricornutum*. Geomicrobiol. J..

[25] Gouda M., Tadda M.A., Zhao Y., Farmanullah F., Chu B., Li X., He Y. (2022). Microalgae Bioactive Carbohydrates as a Novel Sustainable and Eco-Friendly Source of Prebiotics: Emerging Health Functionality and Recent Technologies for Extraction and Detection. Front. Nutr..

[26] Ran W., Wang H., Liu Y., Qi M., Xiang Q., Yao C., Zhang Y., Lan X. (2019). Storage of Starch and Lipids in Microalgae: Biosynthesis and Manipulation by Nutrients. Bioresour. Technol..

[27] Kaur A., Taggar M.S., Kalia A., Singh M. (2022). Nitrate-Induced Carbohydrate Accumulation in *Chlorella sorokiniana* and Its Potential for Ethanol Production. Bioenergy Res..

[28] Manning S.R., Perri K.A., Blackwell K. (2022). Bioactive Polysaccharides from Microalgae. Polysaccharides of Microbial Origin: Biomedical Applications.

[29] Jensen E.L., Yangüez K., Carrière F., Gontero B. (2019). Storage Compound Accumulation in Diatoms as Response to Elevated CO_2_ Concentration. Biology.

[30] Wang F., Yang R., Guo Y., Zhang C. (2022). Isolation, Characterization and Immunomodulatory Activity Evaluation of Chrysolaminarin from the Filamentous Microalga Tribonema Aequale. Mar. Drugs.

[31] Abadi B., Hosseinalipour S., Nikzad S., Pourshaikhali S., Fathalipour-Rayeni H., Shafiei G., Adeli-Sardou M., Shakibaie M., Forootanfar H. (2022). Capping Agents for Selenium Nanoparticles in Biomedical Applications. J. Clust. Sci..

[32] Ghanbariasad A., Taghizadeh S.M., Show P.L., Nomanbhay S., Berenjian A., Ghasemi Y., Ebrahiminezhad A. (2019). Controlled Synthesis of Iron Oxyhydroxide (FeOOH) Nanoparticles Using Secretory Compounds from *Chlorella vulgaris* Microalgae. Bioengineered.

[33] Navarro Gallón S.M., Alpaslan E., Wang M., Larese-Casanova P., Londoño M.E., Atehortúa L., Pavón J.J., Webster T.J. (2019). Characterization and Study of the Antibacterial Mechanisms of Silver Nanoparticles Prepared with Microalgal Exopolysaccharides. Mater. Sci. Eng. C.

[34] Jakhu S., Sharma Y., Sharma K., Vaid K., Dhar H., Kumar V., Singh R.P., Shekh A., Kumar G. (2021). Production and Characterization of Microalgal Exopolysaccharide as a Reducing and Stabilizing Agent for Green Synthesis of Gold-Nanoparticle: A Case Study with a *Chlorella* Sp. from Himalayan High-Altitude Psychrophilic Habitat. J. Appl. Phycol..

[35] Callejón M.J.J., Medina A.R., Sánchez M.D.M., Moreno P.A.G., López E.N., Cerdán L.E., Grima E.M. (2022). Supercritical Fluid Extraction and Pressurized Liquid Extraction Processes Applied to Eicosapentaenoic Acid-Rich Polar Lipid Recovery from the Microalga *Nannochloropsis* Sp.. Algal Res..

[36] Khoo K.S., Ahmad I., Chew K.W., Iwamoto K., Bhatnagar A., Show P.L. (2023). Enhanced Microalgal Lipid Production for Biofuel Using Different Strategies Including Genetic Modification of Microalgae: A Review. Prog. Energy Combust. Sci..

[37] Vrana I., Bakija Alempijević S., Novosel N., Ivošević DeNardis N., Žigon D., Ogrinc N., Gašparović B. (2022). Hyposalinity Induces Significant Polar Lipid Remodeling in the Marine Microalga *Dunaliella tertiolecta* (Chlorophyceae). J. Appl. Phycol..

[38] Zheng G., Gu F., Cui Y., Lu L., Hu X., Wang L., Wang Y. (2022). A Microfluidic Droplet Array Demonstrating High-Throughput Screening in Individual Lipid-Producing Microalgae. Anal. Chim. Acta.

[39] Karimi K., Saidi M., Moradi P., Taheri Najafabadi A. (2023). Biodiesel Production from Nannochloropsis Microalgal Biomass-Derived Oil: An Experimental and Theoretical Study Using the RSM-CCD Approach. Can. J. Chem. Eng..

[40] Kafil M., Berninger F., Koutra E., Kornaros M. (2022). Utilization of the Microalga Scenedesmus Quadricauda for Hexavalent Chromium Bioremediation and Biodiesel Production. Bioresour. Technol..

[41] Kashyap M., Samadhiya K., Ghosh A., Anand V., Shirage P.M., Bala K. (2019). Screening of Microalgae for Biosynthesis and Optimization of Ag/AgCl Nano Hybrids Having Antibacterial Effect. RSC Adv..

[42] Gusain D., Renuka N., Guldhe A., Bux F. (2021). Use of Microalgal Lipids and Carbohydrates for the Synthesis of Carbon Dots via Hydrothermal Microwave Treatment. Inorg. Chem. Commun..

[43] Algal M., Alprol A.E., Tageldein Mansour A., El-Beltagi H.S., Ashour M. (2023). Algal Extracts for Green Synthesis of Zinc Oxide Nanoparticles: Promising Approach for Algae Bioremediation. Materials.

[44] Li X., Mao X., Xie W., Liu B., Chen F. (2022). Intracellular Biosynthesis of Gold Nanoparticles for Monitoring Microalgal Biomass via Surface-Enhanced Raman Spectroscopy. ACS Sustain. Chem. Eng..

[45] Kashyap M., Samadhiya K., Ghosh A., Anand V., Lee H., Sawamoto N., Ogura A., Ohshita Y., Shirage P.M., Bala K. (2021). Synthesis, Characterization and Application of Intracellular Ag/AgCl Nanohybrids Biosynthesized in *Scenedesmus* Sp. as Neutral Lipid Inducer and Antibacterial Agent. Environ. Res..

[46] Zhang Z., Chen J., Yang Q., Lan K., Yan Z., Chen J. (2018). Eco-Friendly Intracellular Microalgae Synthesis of Fluorescent CdSe QDs as a Sensitive Nanoprobe for Determination of Imatinib. Sensors Actuators B Chem..

[47] Yilmaz Öztürk B. (2019). Intracellular and Extracellular Green Synthesis of Silver Nanoparticles Using *Desmodesmus* Sp.: Their Antibacterial and Antifungal Effects. Caryologia.

[48] Hardiningtyas S.D., Putri F.A., Setyaningsih I. (2022). Antibacterial Activity of Ethanolic Spirulina Platensis Extract-Water Soluble Chitosan Nanoparticles. IOP Conf. Ser. Earth Environ. Sci..

[49] Muthusamy G., Thangasamy S., Raja M., Chinnappan S., Kandasamy S. (2017). Biosynthesis of Silver Nanoparticles from *Spirulina* Microalgae and Its Antibacterial Activity. Environ. Sci. Pollut. Res..

[50] Darwesh O.M., Matter I.A., Eida M.F., Moawad H., Oh Y.K. (2019). Influence of Nitrogen Source and Growth Phase on Extracellular Biosynthesis of Silver Nanoparticles Using Cultural Filtrates of *Scenedesmus obliquus*. Appl. Sci..

[51] Rajkumar R., Ezhumalai G., Gnanadesigan M. (2021). A Green Approach for the Synthesis of Silver Nanoparticles by *Chlorella vulgaris* and Its Application in Photocatalytic Dye Degradation Activity. Environ. Technol. Innov..

[52] Shalaby S.M., Madkour F.F., El-Kassas H.Y., Mohamed A.A., Elgarahy A.M. (2021). Green Synthesis of Recyclable Iron Oxide Nanoparticles Using Spirulina Platensis Microalgae for Adsorptive Removal of Cationic and Anionic Dyes. Environ. Sci. Pollut. Res..

[53] Hamida R.S., Ali M.A., Almohawes Z.N., Alahdal H., Momenah M.A., Bin-Meferij M.M. (2022). Green Synthesis of Hexagonal Silver Nanoparticles Using a Novel Microalgae Coelastrella Aeroterrestrica Strain BA_Chlo4 and Resulting Anticancer, Antibacterial, and Antioxidant Activities. Pharmaceutics.

[54] Fani A., Varmazyar S., Akbari F., Garfami M., Mohaghegh R., Balkhi S., Mojdehi S.R., Tabassi N.R., Hosseinpour T., Ghanbari Z. (2023). Green Synthesis of a Novel PtFe2O4@Ag Nanocomposite: Implications for Cytotoxicity, Gene Expression and Anti-Cancer Studies in Gastric Cancer Cell Line. J. Clust. Sci..

[55] Sharif A.P., Habibi K., Bijarpas Z.K., Tolami H.F., Alkinani T.A., Jameh M., Dehkaei A.A., Monhaser S.K., Daemi H.B., Mahmoudi A. (2022). Cytotoxic Effect of a Novel GaFe_2_O_4_@Ag Nanocomposite Synthesized by Scenedesmus Obliquus on Gastric Cancer Cell Line and Evaluation of BAX, Bcl-2 and CASP8 Genes Expression. J. Clust. Sci..

[56] Kardan M., Pouraei A., Jaahbin N., Ghasemipour T., Mehraban F., Jahani Sayyad Noveiri M., Hedayati M., Salehzadeh A. (2022). Cytotoxicity of Bio-Synthesized MgFe2O4@Ag Nanocomposite on Gastric Cancer Cell Line and Evaluation Its Effect on *Bax*, *P53* and *Bcl-2* Genes Expression. J. Clust. Sci..

[57] İnan B., Mutlu B., Karaca G.A., Koç R.Ç., Özçimen D. (2023). Bioprospecting Antarctic Microalgae as Anticancer Agent against PC-3 and AGS Cell Lines. Biochem. Eng. J..

[58] Doman K.M., Gharieb M.M., Abd El-Monem A.M., Morsi H.H. (2023). Synthesis of Silver and Copper Nanoparticle Using Spirulina Platensis and Evaluation of Their Anticancer Activity. Int. J. Environ. Health Res..

[59] Soror A.F.S., Ahmed M.W., Hassan A.E.A., Alharbi M., Alsubhi N.H., Al-Quwaie D.A., Alrefaei G.I., Binothman N., Aljadani M., Qahl S.H. (2022). Evaluation of Green Silver Nanoparticles Fabricated by Spirulina Platensis Phycocyanin as Anticancer and Antimicrobial Agents. Life.

[60] Hamouda R.A., Abd El Maksoud A.I., Wageed M., Alotaibi A.S., Elebeedy D., Khalil H., Hassan A., Abdella A. (2021). Characterization and Anticancer Activity of Biosynthesized Au/Cellulose Nanocomposite from *Chlorella vulgaris*. Polymers.

[61] Singh A.K., Tiwari R., Singh V.K., Singh P., Khadim S.R., Singh U., Laxmi, Srivastava V., Hasan S.H., Asthana R.K. (2019). Green Synthesis of Gold Nanoparticles from Dunaliella Salina, Its Characterization and in Vitro Anticancer Activity on Breast Cancer Cell Line. J. Drug Deliv. Sci. Technol..

[62] Amjad M., Iqbal M., Faisal A., Junjua A.M., Hussain I., Hussain S.Z., Ghramh H.A., Khan K.A., Janjua H.A. (2019). Hydrothermal Synthesis of Carbon Nanodots from Bovine Gelatin and PHM3 Microalgae Strain for Anticancer and Bioimaging Applications. Nanoscale Adv..

[63] Sathishkumar R.S., Sundaramanickam A., Srinath R., Ramesh T., Saranya K., Meena M., Surya P. (2019). Green Synthesis of Silver Nanoparticles by Bloom Forming Marine Microalgae Trichodesmium Erythraeum and Its Applications in Antioxidant, Drug-Resistant Bacteria, and Cytotoxicity Activity. J. Saudi Chem. Soc..

[64] El-Sheekh M.M., Hassan L.H.S., Morsi H.H. (2021). Assessment of the In Vitro Anticancer Activities of Cyanobacteria Mediated Silver Oxide and Gold Nanoparticles in Human Colon CaCo-2 and Cervical HeLa Cells. Environ. Nanotechnol. Monit. Manag..

[65] Huang G., Chen X., Li N., Xie T., Guo Y., Fu Y., Jiao T. (2022). A Convenient Synthesis of Gold Nanoparticles in *Spirulina* Extract for Rapid Visual Detection of Dopamine in Human Urine. Colloids Surf. A Physicochem. Eng. Asp..

[66] Ameen F., Hamidian Y., Mostafazadeh R., Darabi R., Erk N., Islam M.A., Orfali R. (2023). A Novel Atropine Electrochemical Sensor Based on Silver Nano Particle-Coated Spirulina Platensis Multicellular Blue-Green Microalga. Chemosphere.

[67] Jafari S.M., Masoum S., Tafreshi S.A.H. (2021). A Microlagal-Based Carbonaceous Sensor for Enzymatic Determination of Glucose in Blood Serum. J. Ind. Eng. Chem..

[68] Wang X., Cai J., Sun L., Zhang S., Gong D., Li X., Yue S., Feng L., Zhang D. (2019). Facile Fabrication of Magnetic Microrobots Based on Spirulina Templates for Targeted Delivery and Synergistic Chemo-Photothermal Therapy. ACS Appl. Mater. Interfaces.

[69] Li M., Wu J., Lin D., Yang J., Jiao N., Wang Y., Liu L. (2022). A Diatom-Based Biohybrid Microrobot with a High Drug-Loading Capacity and PH-Sensitive Drug Release for Target Therapy. Acta Biomater..

[70] Saxena A., Dutta A., Kapoor N., Kumar A., Tiwari A. (2022). Envisaging Marine Diatom Thalassiosira Weissflogii as a “SMART” Drug Delivery System for Insoluble Drugs. J. Drug Deliv. Sci. Technol..

[71] İnan B., Özçimen D. (2021). Preparation and Characterization of Microalgal Oil Loaded Alginate/Poly (Vinyl Alcohol) Electrosprayed Nanoparticles. Food Bioprod. Process..

[72] Chandrarathna H.P.S.U., Liyanage T.D., Edirisinghe S.L., Dananjaya S.H.S., Thulshan E.H.T., Nikapitiya C., Oh C., Kang D.H., de Zoysa M. (2020). Marine Microalgae, Spirulina Maxima-Derived Modified Pectin and Modified Pectin Nanoparticles Modulate the Gut Microbiota and Trigger Immune Responses in Mice. Mar. Drugs.

[73] Rajapaksha D.C., Edirisinghe S.L., Nikapitiya C., Dananjaya S.H.S., Kwun H.J., Kim C.H., Oh C., Kang D.H., De Zoysa M. (2020). Spirulina Maxima Derived Pectin Nanoparticles Enhance the Immunomodulation, Stress Tolerance, and Wound Healing in Zebrafish. Mar. Drugs.

[74] El-Deeb N.M., Abo-Eleneen M.A., Al-Madboly L.A., Sharaf M.M., Othman S.S., Ibrahim O.M., Mubarak M.S. (2020). Biogenically Synthesized Polysaccharides-Capped Silver Nanoparticles: Immunomodulatory and Antibacterial Potentialities Against Resistant *Pseudomonas aeruginosa*. Front. Bioeng. Biotechnol..

[75] Roy A., Bulut O., Some S., Mandal A.K., Yilmaz M.D. (2019). Green Synthesis of Silver Nanoparticles: Biomolecule-Nanoparticle Organizations Targeting Antimicrobial Activity. RSC Adv..

[76] Khalid M., Khalid N., Ahmed I., Hanif R., Ismail M., Janjua H.A. (2017). Comparative Studies of Three Novel Freshwater Microalgae Strains for Synthesis of Silver Nanoparticles: Insights of Characterization, Antibacterial, Cytotoxicity and Antiviral Activities. J. Appl. Phycol..

[77] Suganya K.S.U., Govindaraju K., Kumar V.G., Dhas T.S., Karthick V., Singaravelu G., Elanchezhiyan M. (2015). Size Controlled Biogenic Silver Nanoparticles as Antibacterial Agent against Isolates from HIV Infected Patients. Spectrochim. Acta Part A Mol. Biomol. Spectrosc..

[78] Omomowo I.O., Adenigba V.O., Ogunsona S.B., Adeyinka G.C., Oluyide O.O., Adedayo A.A., Fatukasi B.A. (2020). Antimicrobial and Antioxidant Activities of Algal-Mediated Silver and Gold Nanoparticles. IOP Conf. Ser. Mater. Sci. Eng..

[79] Jena J., Pradhan N., Dash B.P., Sukla L.B., Panda P.K. (2013). Biosynthesis and Characterization of Silver Nanoparticles Using Microalga *Chlorococcum humicola* and Its Antibacterial Activity. Int. J. Nanomater. Biostruct..

[80] Jena J., Pradhan N., Nayak R.R., Dash B.P., Sukla L.B., Panda P.K., Mishra B.K. (2014). Microalga Scenedesmus Sp.: A Potential Low-Cost Green Machine for Silver Nanoparticle Synthesis. J. Microbiol. Biotechnol..

[81] Ebrahiminezhad A., Bagheri M., Taghizadeh S.M., Berenjian A., Ghasemi Y. (2016). Biomimetic Synthesis of Silver Nanoparticles Using Microalgal Secretory Carbohydrates as a Novel Anticancer and Antimicrobial. Adv. Nat. Sci. Nanosci. Nanotechnol..

[82] Sahoo C.R., Maharana S., Mandhata C.P., Bishoyi A.K., Paidesetty S.K., Padhy R.N. (2020). Biogenic Silver Nanoparticle Synthesis with Cyanobacterium *Chroococcus minutus* Isolated from Baliharachandi Sea-Mouth, Odisha, and in Vitro Antibacterial Activity. Saudi J. Biol. Sci..

[83] Hamouda R.A., Hussein M.H., Abo-elmagd R.A., Bawazir S.S. (2019). Synthesis and Biological Characterization of Silver Nanoparticles Derived from the Cyanobacterium *Oscillatoria limnetica*. Sci. Rep..

[84] Bishoyi A.K., Sahoo C.R., Sahoo A.P., Padhy R.N. (2021). Bio-Synthesis of Silver Nanoparticles with the Brackish Water Blue-Green Alga *Oscillatoria princeps* and Antibacterial Assessment. Appl. Nanosci..

[85] Patel V., Berthold D., Puranik P., Gantar M. (2015). Screening of Cyanobacteria and Microalgae for Their Ability to Synthesize Silver Nanoparticles with Antibacterial Activity. Biotechnol. Rep..

[86] Aziz N., Faraz M., Pandey R., Shakir M., Fatma T., Varma A., Barman I., Prasad R. (2015). Facile Algae-Derived Route to Biogenic Silver Nanoparticles: Synthesis, Antibacterial, and Photocatalytic Properties. Langmuir.

[87] Salari Z., Danafar F., Dabaghi S., Ataei S.A. (2016). Sustainable Synthesis of Silver Nanoparticles Using Macroalgae *Spirogyra varians* and Analysis of Their Antibacterial Activity. J. Saudi Chem. Soc..

[88] Taghizadeh S.M., Lal N., Ebrahiminezhad A., Moeini F., Seifan M., Ghasemi Y., Berenjian A. (2020). Green and Economic Fabrication of Zinc Oxide (ZnO) Nanorods as a Broadband UV Blocker and Antimicrobial Agent. Nanomaterials.

[89] El-Belely E.F., Farag M.M.S., Said H.A., Amin A.S., Azab E., Gobouri A.A., Fouda A. (2021). Green Synthesis of Zinc Oxide Nanoparticles (ZnO-NPs) Using Arthrospira Platensis (Class: Cyanophyceae) and Evaluation of Their Biomedical Activities. Nanomaterials.

[90] Martins-Santana L., Rezende C.P., Rossi A., Martinez-Rossi N.M., Almeida F. (2023). Addressing Microbial Resistance Worldwide: Challenges over Controlling Life-Threatening Fungal Infections. Pathogens.

[91] Scaglioni P.T., Pagnussatt F.A., Lemos A.C., Nicolli C.P., Del Ponte E.M., Badiale-Furlong E. (2019). *Nannochloropsis* Sp. and *Spirulina* Sp. as a Source of Antifungal Compounds to Mitigate Contamination by Fusarium Graminearum Species Complex. Curr. Microbiol..

[92] Sidorowicz A., Margarita V., Fais G., Pantaleo A., Manca A., Concas A., Rappelli P., Fiori P.L., Cao G. (2022). Characterization of Nanomaterials Synthesized from *Spirulina platensis* Extract and Their Potential Antifungal Activity. PLoS ONE.

[93] Gürsoy N., Yilmaz Öztürk B., Dağ İ. (2021). Synthesis of Intracellular and Extracellular Gold Nanoparticles with a Green Machine and Its Antifungal Activity. Turk. J. Biol..

[94] Annamalai J., Nallamuthu T. (2015). Characterization of Biosynthesized Gold Nanoparticles from Aqueous Extract of *Chlorella vulgaris* and Their Anti-Pathogenic Properties. Appl. Nanosci..

[95] Win T.T., Khan S., Bo B., Zada S., Fu P.C. (2021). Green Synthesis and Characterization of Fe_3_O_4_ Nanoparticles Using Chlorella-K01 Extract for Potential Enhancement of Plant Growth Stimulating and Antifungal Activity. Sci. Rep..

[96] Torres-Díaz M., Abreu-Takemura C., Díaz-Vázquez L.M. (2022). Microalgae Peptide-Stabilized Gold Nanoparticles as a Versatile Material for Biomedical Applications. Life.

[97] Rudi L., Zinicovscaia I., Cepoi L., Chiriac T., Peshkova A., Cepoi A., Grozdov D. (2021). Accumulation and Effect of Silver Nanoparticles Functionalized with Spirulina Platensis on Rats. Nanomaterials.

[98] Liu C., Fu Y., Li C.E., Chen T., Li X. (2017). Phycocyanin-Functionalized Selenium Nanoparticles Reverse Palmitic Acid-Induced Pancreatic β Cell Apoptosis by Enhancing Cellular Uptake and Blocking Reactive Oxygen Species (ROS)-Mediated Mitochondria Dysfunction. J. Agric. Food Chem..

[99] Yang F., Tang Q., Zhong X., Bai Y., Chen T., Zhang Y., Li Y., Zheng W. (2012). Surface Decoration by *Spirulina polysaccharide* Enhances the Cellular Uptake and Anticancer Efficacy of Selenium Nanoparticles. Int. J. Nanomed..

